# SARS-CoV-2 antibodies in Colombian urban area: follow-up on occupational groups

**DOI:** 10.15649/cuidarte.4029

**Published:** 2025-07-28

**Authors:** Claudia C. Colmenares-Mejía, Doris C. Quintero-Lesmes, Norma C. Serrano, Ligia Meneses, Isail Salazar Acosta, Álvaro J. Idrovo, Duván Y. Sanabria-Echeverry, Helmer Cordero Rebolledo, Víctor Castillo

**Affiliations:** 1 Research Centre, Fundación Cardiovascular de Colombia. Floridablanca, Colombia. E.mail: claudiacolmenares@fcv.org Fundación Cardiovascular de Colombia Floridablanca Colombia claudiacolmenares@fcv.org; 2 Research Centre, Fundación Cardiovascular de Colombia. Floridablanca, Colombia. E-mail: dorisquintero@fcv.org Fundación Cardiovascular de Colombia Floridablanca Colombia dorisquintero@fcv.org; 3 Research Centre, Fundación Cardiovascular de Colombia. Floridablanca, Colombia. E-mail: normaserrano@fcv.org Fundación Cardiovascular de Colombia Floridablanca Colombia normaserrano@fcv.org; 4 Clinical Laboratory, Fundación Cardiovascular de Colombia. Floridablanca, Colombia. E-mail: ligiameneses@fcv.org Fundación Cardiovascular de Colombia Floridablanca Colombia ligiameneses@fcv.org; 5 Research Centre, Fundación Cardiovascular de Colombia. Floridablanca, Colombia. E.mail: isailsalazar@fcv.org Fundación Cardiovascular de Colombia Floridablanca Colombia isailsalazar@fcv.org; 6 Public Health Department, Universidad Industrial de Santander. Bucaramanga, Colombia. E-mail: idrovoaj@uis.edu.co Universidad Industrial de Santander Bucaramanga Colombia idrovoaj@uis.edu.co; 7 School of Civil Engineering, Universidad Industrial de Santander. Bucaramanga, Colombia. E-mail: duvansan@uis.edu.co Universidad Industrial de Santander Bucaramanga Colombia duvansan@uis.edu.co; 8 School of Civil Engineering, Universidad Industrial de Santander. Bucaramanga, Colombia. E-mail: helmercordero@gmail.com Universidad Industrial de Santander Bucaramanga Colombia helmercordero@gmail.com; 9 Fundación Cardiovascular de Colombia, Floridablanca, Colombia. E-mail: victorcastillo@fcv.org Fundación Cardiovascular de Colombia Floridablanca Colombia victorcastillo@fcv.org

**Keywords:** COVID-19, COVID-19 Serological Testing, Occupational Health, COVID-19, Prueba Serológica para COVID-19, Salud Ocupacional, COVID-19, Teste Sorológico para COVID-19, Saúde Ocupacional

## Abstract

**Introduction:**

Globally, the COVID-19 pandemic typically relied on reported cases, but a more comprehensive view emerges from antibody identification. A 2020 national study in Bucaramanga revealed diverse SARS-CoV-2 exposure levels among occupational groups, emphasizing the vulnerability of informal workers.

**Objective:**

This study estimates adjusted seroprevalence and seroincidence of SARS-CoV-2 antibodies (IgM and IgG) among occupational groups in the Bucaramanga Metropolitan Area (Colombia) in three different moments of the pandemic.

**Materials and Methods:**

The prospective cohort study was conducted from September 2020 to October 2021, comprising three rounds. Each round gathered demographic data, medical history, COVID exposure or diagnosis, and vaccination status from workers across various occupational groups in Bucaramanga and its metropolitan area. Immunoglobulins G and M were assessed in each round, and adjusted seroprevalence was calculated.

**Results:**

A total of 1,013 subjects participated in all rounds. Healthcare workers accounted for more than 50% of all participants. By Round 3, 93.6% (n = 948) of participants had received the vaccination. Adjusted seroprevalence increased progressively (Round 1: 15.9% [95%CI 13.7–18.2]; Round 2: 18.4% [95%CI 16.0–20.7]; Round 3: 29.2% [95%CI 26.4–31.9]). Seroincidence for IgM/IgG positivity by Round 3 was 26.2% (95%CI 20.7–31.6).

**Discussion:**

The progressive increase in adjusted SARS-CoV-2 seroprevalence among workers in the Metropolitan Area of Bucaramanga is highlighted, with a notable prevalence among healthcare workers.

**Conclusion:**

The study underscores the prevalence of SARS-CoV-2 infection among workers in the Bucaramanga Metropolitan Area, emphasizing the role of hybrid immunity following vaccination in infection rates. Continued monitoring and tailored interventions are vital for protecting vulnerable populations.

## Resumen

**Introducción:**

A nivel mundial, la pandemia de COVID-19 generalmente se basa en casos reportados, pero una visión más integral surge de la identificación de anticuerpos. Un estudio nacional de 2020 en Bucaramanga reveló diversos niveles de exposición al SARS-CoV-2 entre grupos ocupacionales, enfatizando la vulnerabilidad de los trabajadores informales.

**Objetivo:**

Este estudio estima la seroprevalencia ajustada y la seroincidencia de anticuerpos contra el SARS-CoV-2 (IgM e IgG) entre grupos ocupacionales del Área Metropolitana de Bucaramanga (Colombia) en tres momentos diferentes de la pandemia.

**Materiales y métodos:**

El estudio de cohorte prospectivo, realizado de septiembre de 2020 a octubre de 2021, comprendió tres rondas. Cada ronda recopiló datos demográficos, historial médico, exposición o diagnóstico de COVID y estado de vacunación de empleados de varios grupos ocupacionales en Bucaramanga y su área metropolitana. Se evaluaron las inmunoglobulinas G y M en cada ronda, y se calculó la seroprevalencia ajustada.

**Resultados:**

1.013 sujetos participaron en todas las rondas. Los trabajadores de la salud representaron más del 50% de todos los participantes. El 93,6% (n = 948) de los participantes estaban vacunados para la tercera ronda. La seroprevalencia ajustada aumentó progresivamente [Ronda 1: 15,9% (IC del 95% 13,7–18,2); Ronda 2: 18,4% (IC del 95% 16,0–20,7); Ronda 3: 29,2% (IC del 95% 26,4–31,9)]. La seroincidencia para IgM/IgG positivo para la tercera ronda fue del 26,2% (IC del 95% 20,7–31,6).

**Discusión:**

Se destaca el aumento progresivo de la seroprevalencia ajustada de anticuerpos contra el SARS-CoV-2 entre los trabajadores del Área Metropolitana de Bucaramanga, con una notable prevalencia entre los trabajadores de la salud.

**Conclusiones:**

El estudio destaca la prevalencia de infecciones por SARS-CoV-2 entre los trabajadores del Área Metropolitana de Bucaramanga, enfatizando el papel de la inmunidad híbrida sobre la vacunación en las tasas de infección. El monitoreo continuo y las intervenciones adaptadas son vitales para proteger a las poblaciones vulnerables.

## Resumo

**Introdução:**

Globalmente, a pandemia de COVID-19 normalmente depende de casos relatados, mas uma visão mais abrangente surge da identificação de anticorpos. Um estudo nacional de 2020 em Bucaramanga revelou diversos níveis de exposição ao SARS-CoV-2 entre grupos ocupacionais, enfatizando a vulnerabilidade de trabalhadores informais.

**Objetivo:**

Este estudo estima a soroprevalência ajustada e a soroincidência de anticorpos SARS-CoV-2 (IgM e IgG) entre grupos ocupacionais da Área Metropolitana de Bucaramanga (Colômbia) em três momentos diferentes da pandemia.

**Materiais e métodos:**

O estudo de coorte prospectivo, conduzido de setembro de 2020 a outubro de 2021, compreendeu três rodadas. Cada rodada coletou dados demográficos, histórico médico, exposição ou diagnóstico de COVID e status de vacinação de funcionários de vários grupos ocupacionais em Bucaramanga e sua área metropolitana. As imunoglobulinas G e M foram avaliadas em cada rodada, e a soroprevalência ajustada foi calculada.

**Resultados:**

1.013 indivíduos participaram de todas as rodadas. Os profissionais de saúde representaram mais de 50% de todos os participantes. 93,6% (n=948) dos participantes foram vacinados na rodada 3. A soroprevalência ajustada aumentou progressivamente [rodada 1: 15,9% (IC95% 13,7–18,2); rodada 2: 18,4% (IC95% 16,0–20,7); rodada 3: 29,2% (IC95% 26,4–31,9)]. A soroincidência para IgM/IgG positivo na rodada 3 foi de 26,2% (IC95% 20,7–31,6).

**Discussão:**

O aumento progressivo na soroprevalência ajustada de anticorpos contra SARS-CoV-2 entre os trabalhadores da Área Metropolitana de Bucaramanga é destacado, com uma prevalência notável entre os profissionais de saúde.

**Conclusões:**

O estudo ressalta a prevalência de infecções por SARS-CoV-2 entre trabalhadores da Área Metropolitana de Bucaramanga, enfatizando o papel da imunidade híbrida sobre a vacinação nas taxas de infecção. Monitoramento contínuo e intervenções personalizadas são vitais para proteger populações vulneráveis.

## Introduction

The evolution of the COVID-19 pandemic was usually monitored through the number of infected individuals, hospitalizations, and deaths. However, this conventional approach was incomplete, showing only the tip of the iceberg. One way to complement this information was through the identification of infected individuals by detecting antibodies^1^. This method allows a better understanding of the situation by identifying asymptomatic individuals. Many seroprevalence studies worldwide have shown the magnitude of the pandemic at national, regional, and local levels, as well as within specific population groups^2^. Although each country experienced the evolution of the pandemic differently, Colombia presents an interesting case for analysis due to several unique events. 

Noteworthy among these were various instances of economic reopening, which were often followed by surges in case numbers. Additionally, Colombia experienced prolonged lockdown periods, including school closures, as well as enduring social protests, the most sustained observed during the pandemic^3^–^5^. These events contributed to a reduced perception of risk among different social groups^6^, marking significant milestones in the country's history during this time.

A national seroprevalence study conducted in 2020 identified great heterogeneity in infections after the first wave (approximately June to September 2020) across different cities studied^7^. However, no subsequent follow-ups were conducted to monitor the pandemic's evolution during or after the three subsequent waves (approximately December 2020 to February 2021, April to August 2021, and January to February 2022)^3^. In Colombia, only the Bucaramanga Metropolitan Area carried out continued surveillance by measuring Immunoglobulin M (IgM) and Immunoglobulin G (IgG) among different occupational groups during these periods. This approach enables the identification of differential exposure levels to SARS-CoV-2 by occupation, underscoring the high vulnerability of informal workers.

Unlike previous studies that offered cross-sectional estimates of seroprevalence, our study addresses the critical gap in longitudinal data by tracking the serological response of occupational groups over time. This approach provides a more detailed understanding of infection dynamics, particularly among essential and informal workers who have often been underrepresented in global seroprevalence studies. Furthermore, our findings have direct public health implications, highlighting occupational disparities in exposure and underscoring the need for workplace safety measures while informing future pandemic preparedness policies. 

This study aimed to estimate the presence of SARS-CoV-2 antibodies (IgM and IgG) among occupational groups in the Bucaramanga Metropolitan Area (Colombia) at three different points during the pandemic and to estimate the adjusted seroincidence of SARS-CoV-2 infection between 3 and 12 months after the first assessment. A follow-up to the same occupational groups is important to identify the potential effects of political decisions and propose improvements for managing future public health crises across different types of workers.

Policymakers should prioritize the protection of vulnerable occupational groups, such as informal workers, and reinforce workplace safety measures. Targeted public health campaigns are also needed to address pandemic-related challenges, enhance risk perception, and encourage adherence to preventive measures. Future research should include longitudinal monitoring of infection rates and immunity across diverse population groups, assess the effectiveness of policy interventions, and explore the socioeconomic determinants of COVID-19 disparities. Moreover, research should delve into the influence of political decisions and social events on public perceptions, assess the long-term effects of lockdown measures, examine the cultural influences on risk behaviors, and evaluate cross-sectoral collaborations for pandemic preparedness and resilience in Colombia.

## Materials and Methods

**Study Design and Population**

An observational cohort study was conducted in the Bucaramanga Metropolitan Area with data collection carried out at three different time points: Round 1 (September 28 to December 24, 2020), Round 2 (December 25, 2020 to March 4, 2021), and Round 3 (August 28 to October 15, 2021). To reduce the probability of participant attrition, at least two telephone numbers and an email address were collected for each participant. Bucaramanga, a city located in the department of Santander in northeastern Colombia, was selected as the study setting. Its metropolitan area includes several municipalities, such as Floridablanca, Giron, and Piedecuesta, which span a geographically diverse region of mountains and valleys, offering a unique context for studying the effects of COVID-19. 

This region has approximately 1,111,999 inhabitants, according to the last National Population and Housing Census carried out in 2018^8^. Bucaramanga was chosen due to its importance as an urban center in Colombia and the sizable population of its metropolitan area, which offers a representative sample for studying virus transmission and immune responses across various occupational groups.

Adults (>18 years old) who had resided in the Bucaramanga Metropolitan Area since August 2020 and were formally employed in any of the following occupational groups were invited to participate: healthcare, construction, public transportation (bus and taxi drivers), military and law enforcement personnel (army, police, and transit officers), and bike couriers. Self-employed and informal workers (including shopkeepers in grocery stores) were also invited, as described in a previous study^9^. These occupational groups were prioritized because they continued working despite the nationwide mobility restrictions in place at the time.

**Sampling Methods**

For formal employment, stratified sampling was conducted by occupational groups based on data from the Bucaramanga Chamber of Commerce. All legally registered companies within the selected occupational categories were identified, and those selected were invited to participate in the study. If a company agreed, study information was sent and distributed among its employees to encourage voluntary participation. For informal workers, convenience sampling was conducted in public markets, grocery stores, and neighborhoods with a high proportion of confirmed COVID-19 cases. This sampling approach was selected due to the difficulty in accessing a complete sampling frame of informal workers, who, by definition, are not registered with any formal entity. 

The selected locations were chosen based on their high concentration of informal workers, thereby increasing the likelihood of recruitment. All participants from Round 1 were invited to participate in the subsequent two rounds, either through their employer's human resource office or via direct contact by phone or email.

**Data Collection and Variables**

All participants self-completed an online survey, as previously described^9^. It included information on sociodemographic characteristics, occupational sector (healthcare, public transportation, military and law enforcement, public utilities, private security, construction, food, education, grocery stores/informal commerce, self-employed workers, administrative/municipal services, cleaning services, bike couriers, or other); cigarette smoking status, medical history, contact with suspected or confirmed COVID-19 cases; presence of COVID-related symptoms; possible exposure to infection based on means of transportation used to go to work or to attend medical appointments; use of personal protective equipment; preventive behaviors, lockdown compliance, history of RT-PCR or rapid diagnostic testing; and hospitalizations or intensive care unit (ICU) stays due to COVID-19. Given that Colombia began its SARS-CoV-2 vaccination on February 18, 2021^10^, additional vaccination-related variables are included in Round 3 (willingness to be vaccinated, vaccinated status, number of doses received, vaccine manufacturer, and any reported side effects). 

Data were collected and managed using REDCap (Research Electronic Data Capture), an electronic data capture platform hosted by Fundación Cardiovascular^11^,^12^. REDCap was selected as the primary data collection tool for its secure, web-based interface, which facilitates standardized data entry, minimizes input errors, and ensures traceability through audit trails. Its features, such as automated validation and real-time monitoring, improved data integrity and facilitated participant follow-up across all study rounds. Electronic informed consent was obtained from all subjects involved in the study prior to data and blood sample collection for each round. This consent form was available for download and personal recordkeeping. No financial incentives were provided for participation.

**Geolocation**

Participants’ addresses were collected in a parameterized format and then standardized according to a street type or intersection using the World Geocoding Service in ArcGIS Online. This service offers Level 2 or good quality geocoding for Colombia, meaning it provides a high degree of street-level address coverage throughout the country^13^. ArcGIS was selected for geolocation processing due to its high-accuracy geocoding capabilities, enabling precise spatial analysis of participant distribution and mobility patterns. This approach enabled the assessment of geographic clustering, potential exposure risks, and changes in residence, thereby reducing misclassification bias and improving the contextual interpretation of infection dynamics.

**Supplemental Figure S1:**
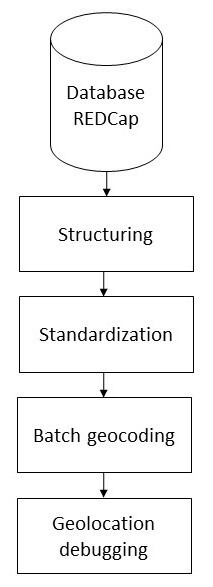
Geolocation process

The geolocation process followed these steps: First, REDCap database was converted into an ArcGIS geodatabase file; second, each address attribute (neighborhood, city, department, and country) was matched against the corresponding fields in the ArcGIS World Composite Geocoder; third, a batch geocoding process was executed to establish real geographic position, converting address data into point-based geographic coordinates on the map); finally, a spatial database was generated, assigning a match score between 0 and 100 to each record, where 100 indicated the highest address accuracy. For records with scores <100, geolocation was debugged manually, using base maps in ArcGIS Online and geographic information systems (Supplemental Figure S1). Changes in home address between Rounds 1 and 3 were assessed for each municipality, considering linear displacements of ≥ 200 meters.

**IgG and IgM Measurement**

For each participant in every study round, a 5 ml peripheral blood sample was obtained by venipuncture in the forearm. Samples were transported from the collection site to the clinical laboratory at Fundación Cardiovascular de Colombia, where IgG antibodies were measured using a chemiluminescence immunoassay, and IgM antibodies were measured by enzymatic fluorescence immunoassay. The Architect SARS-CoV-2 IgG assay (Abbot®) was used for immunoglobulin assessment. This test provides a qualitative result (positive or negative for each antibody). Positive results, whether IgG only, IgM only, or both, were reported to SISMUESTRAS (https://apps.ins.gov.co/sismuestras), the national surveillance platform of the Instituto Nacional de Salud (INS), as a complementary measure for possible case identification, considering the high underestimation of COVID-19 cases reported in the Bucaramanga Metropolitan Area and Colombia^14^. Participants who tested positive for IgM were immediately informed through the email address provided in the online survey. Additionally, for formally employed individuals, the result was reported to their companies' Health and Safety at Work Department to assess the need for confirmatory RT-PCR testing.

**Data Set**

The validated dataset was stored in GitLab^15^.

**Bias Control**

To mitigate potential sources of bias, several strategies were implemented to include participants. Selection bias associated with voluntary participation was addressed by using stratified sampling for formally employed groups based on the Bucaramanga Chamber of Commerce census data. Informal workers were recruited using convenience sampling in areas with high COVID-19 prevalence to enhance the representativeness of the sample. The risk of attrition bias was reduced by collecting at least two phone numbers and an email address for each participant, ensuring follow-up across all study rounds. Geocoding was used to monitor geographic mobility, reducing misclassification bias. 

**Statistical Methods**

**Supplemental Figure S2:**
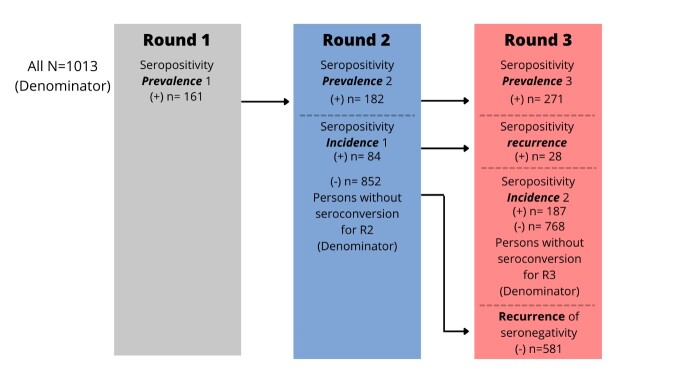
Definitions for Immunological response dynamics

Variables are reported as medians and interquartile ranges (IQR), as well as absolute and relative frequencies. Seroprevalence was estimated as the number of participants with a positive result (for IgG, IgM, or both) divided by the total number of participants and is reported for each of the three rounds. Seroincidence (SI) was defined as a change in antibody status from negative to positive (IgG, IgM, or both) between Round 1 and Round 2 (SI 1) or between Round 2 and Round 3 (SI 2). We also defined seropositivity recurrence (SPR) as the persistence of a positive result between Round 1 and Round 2 (SPR1) and between Round 2 and Round 3 (SPR2). Finally, seronegativity recurrence (SNR) was defined as participants who consistently tested negative for IgM and IgG across all three rounds (Supplemental Figure S2).

The dataset was specified as a complex survey design (svyset). Probability weights (pweights) were calculated by municipality using the formula N/n, where N represents the number of people aged 18 to 85 years, and n is the number of participants in our sample. Stratification was based on occupational sectors. A finite population correction (FPC) was also estimated using √((N − n)/(N − 1)), where N represents the number of people aged 18 and 85 years, and n is the number of participants in the sample. Primary sampling units (PSUs) were defined as the municipalities of Bucaramanga, Floridablanca, Girón, and Piedecuesta). Additionally, frequencies were adjusted for test performance characteristics (sensitivity, 85.2%; specificity, 97.3%) using the correction formula proposed by Sempos and Tian; adjusted prevalence = (crude prevalence + specificity – 1) / (sensitivity + specificity – 1)^16^. 

All analyses were conducted using Stata 15 statistical software (StataCorp., College Station, TX, USA).

**Institutional Review Board Statement**

The study was conducted in accordance with the principles of the Declaration of Helsinki and was approved by the Ethics Committee of Fundación Cardiovascular de Colombia (protocol code CEI-2020-01485, dated September 17, 2020).

## Results

**Figure 1:**
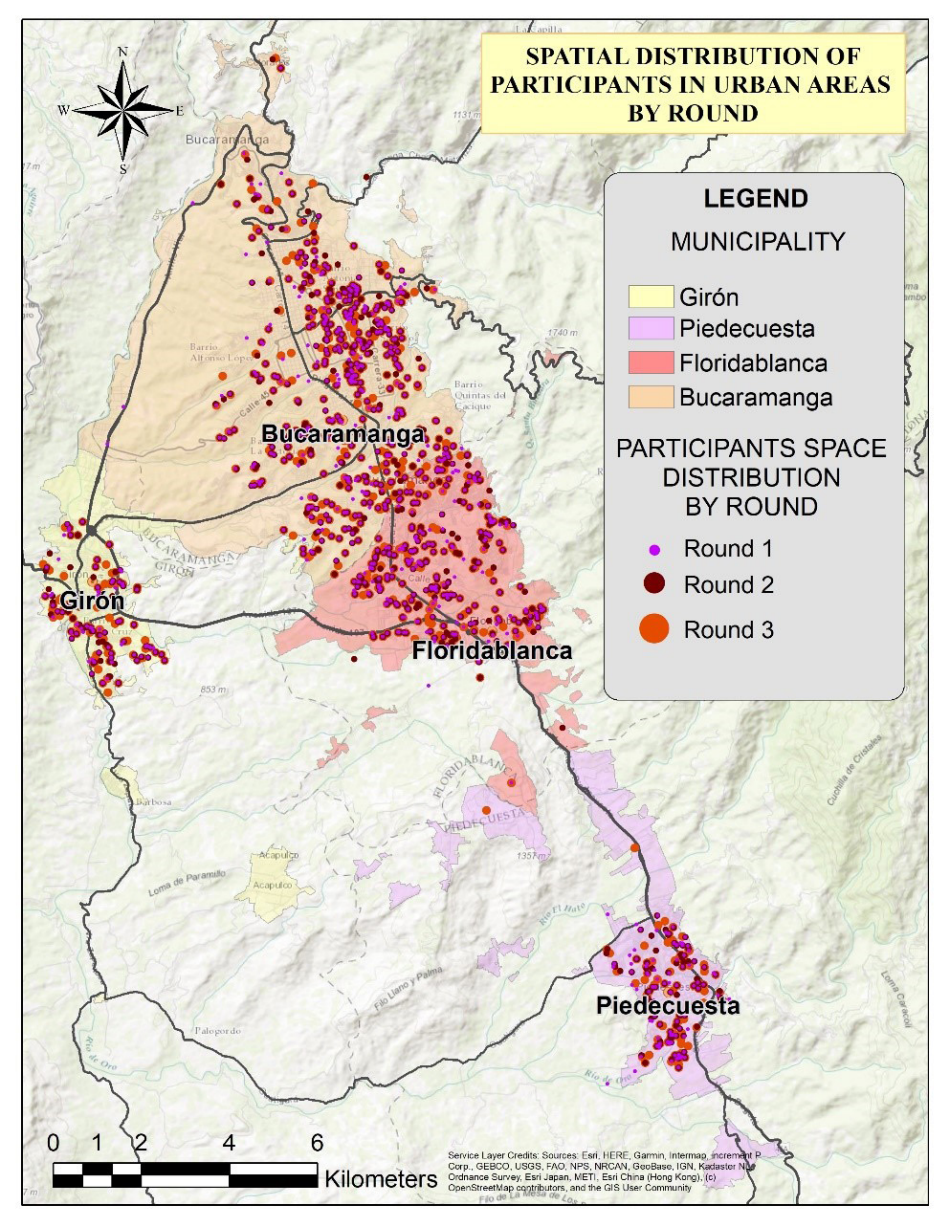
Spatial distribution of participants by rounds

A total of 1,013 participants completed all three rounds of the study. Their spatial distribution is shown in Figure 1. The sample was predominantly composed of women, and the population belonged mainly to a low socioeconomic status (below three). Healthcare workers accounted for more than 50% of all participants (Table 1). 

Likewise, more than 70% of workers reported no pre-existing medical conditions, and only 28.73% had previously received a positive RT-PCR test result for COVID-19 (Table 2). The hospitalization rate due to COVID-19 by round 3 was 0.5%.

**Supplemental Figure S3:**
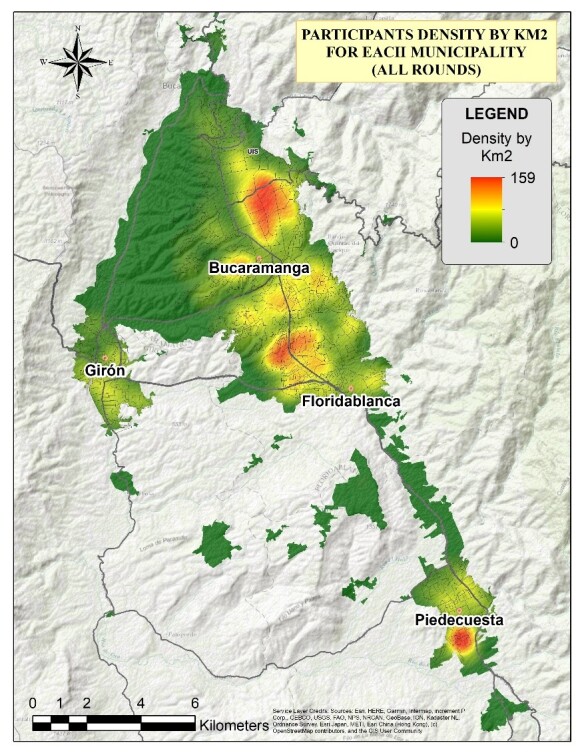
Participant density map for Bucaramanga and its metropolitan area

**Supplemental Figure S4:**
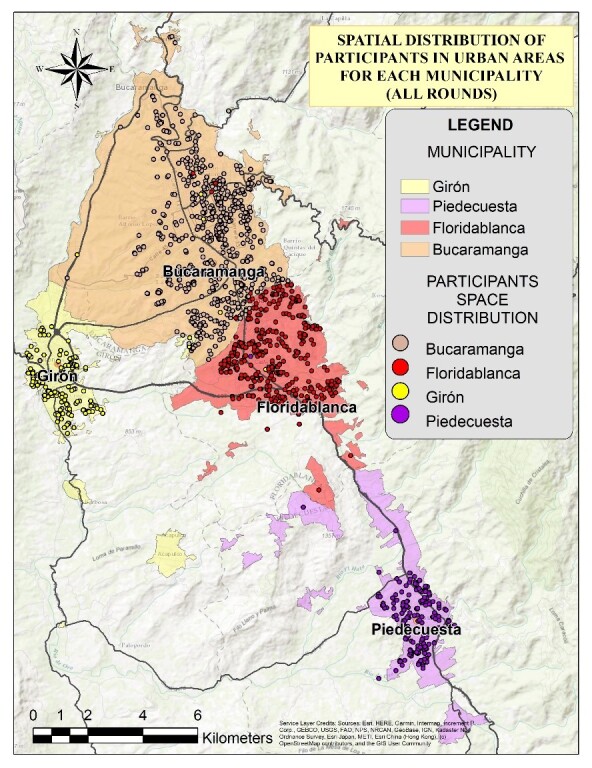
Spatial distribution of participants in Bucaramanga and its metropolitan area

**Supplemental Figure S5:**
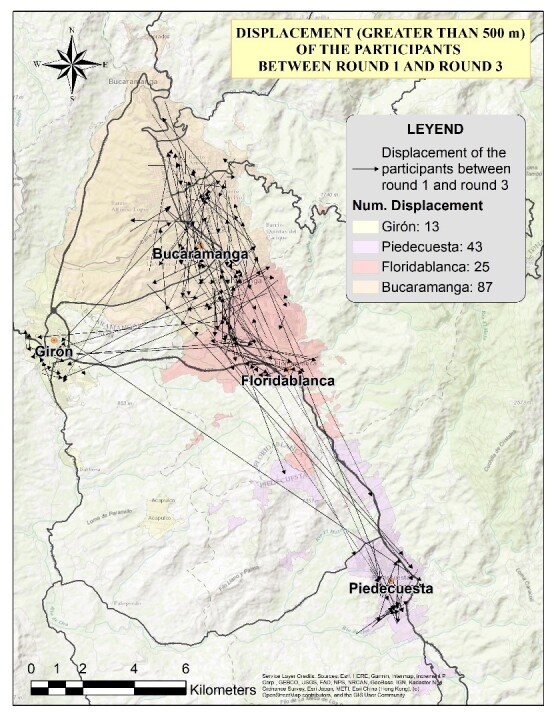
Participant residential displacement between Rounds 1 and 3

Participant density map (Supplemental Figure S3) and spatial distribution (Supplemental Figure S4) for all three rounds are detailed in the supplemental material. Spatial distribution by round is presented in Figure 1. In the geospatial analysis, 168 participants (16.58%) were identified as having changed their residence during the study period, with the majority of these relocations occurring within Bucaramanga (51.47%), followed by Piedecuesta (25.59%), Floridablanca (14.88%), and Girón (7.7%) (Supplemental Figure S4). Although most housing changes occurred within the same municipality, Bucaramanga had the highest number of outbound relocations to other municipalities (Supplemental Figure S5). This displacement may be associated with a decrease in the purchasing power of participants who changed their housing. 

Adjusted seroprevalence increased by each round. In Round 1, adjusted seroprevalence was 16.00% (95% CI: 13.74 – 18.25). This rose to 18.42% (95% CI: 16.06 – 20.78) in Round 2, and, finally, in Round 3, the seroprevalence further increased to 29.21% (95% CI: 26.48 – 31.93). The most marked increases were observed in the municipalities of Girón and Piedecuesta (Figure 2a and 2b. Adjusted Seroprevalence Increase Across Rounds and Municipalities). By group age, participants aged between 40 and 59 years old presented the highest seroprevalence across all rounds, especially in Round 3. Nonetheless, all age groups exhibit an increase in seropositivity over time (Figure 3). By occupational groups, the greatest variations in adjusted seroprevalence by Round 3 (above 60%) were observed among education and security sector workers (Figure 4). Participants who reported a previous positive RT-PCR result had higher seroprevalence, especially in Round 3 (Table 2). The proportion of participants with adjusted SPR1 and SI2 was also higher, especially among participants who tested positive for IgG only (Table 3). The proportion of participants with SNR was 66.18% (95% CI: 62.59 – 70.20).

**Supplemental Figure S6:**
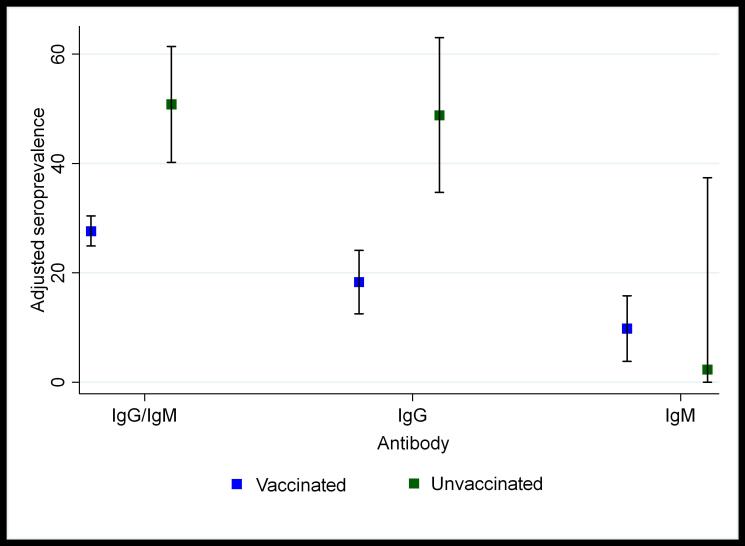
Adjusted seroprevalence by vaccination status in Round 3

For Round 3, vaccination-related information was collected. At that time, 93.58% (n=948) of participants had been vaccinated, and most of them had completed their vaccination schedule (one dose n=191 [20.14%] and two doses n=755 [79.64%]. Two participants did not report vaccination status. The most commonly administered vaccine was BNT162b2 (Pfizer-BioNTech), received by 72.41% of participants, followed by mRNA-1273 (Moderna) at 12.26%, CoronaVac (Sinovac) at 7.93%, and Ad26.COV2.S (Janssen) at 6.66%. Only 0.63% of participants were vaccinated with ChAdOx1-S (Oxford-AstraZeneca). Regarding side effects, 35.94% of vaccinated participants reported experiencing at least one post-vaccination symptom. The most frequently reported were headache (100%), myalgia (29.71%), injection-site pain (23.59%), and fatigue (23.00%). Arthralgias (13.13%), chills (12.73%), fever (10.27%), nausea (6.42%), and vomiting (1.58%) were less frequently reported. One participant reported an anaphylactic reaction following the administration of the CoronaVac (Sinovac) vaccine. Among those who were not vaccinated (n = 65), 32.31% reported that they did not intend to receive the vaccine in the future. Adjusted seroprevalence was lower in vaccinated participants for positive IgG [18.30% (95% CI: 12.53 - 24.07) vs 48.84% (95% CI: 34.68 – 63.01)] and positive IgM/IgG [27.63% (95% CI: 24.86 - 30.41) vs 50.78% (95% CI: 38.70 – 62.87)]. However, it was higher for positive IgM [9.81% (CI95% 3.82 - 15.81) vs 2.30% (95% CI: 0 - 37.41)] (Supplemental Figure S6).

**Table 1:** General characteristics of patients treated in the ICU during the first semester of 2023

Variable	All n=1013 n%	Seropositivity		
		Round 1 % (95% CI)	Round 2 % (95% CI)	Round 3 % (95% CI)
Age (years)*	37 (30 – 44)			
Sex				
Men	357 (35.24)	17.69 (13.92 – 21.46)	23.39 (18.93 – 27.84)	34.30 (29.51 – 39.09)
Women	656 (64.76)	15.03 (12.29 – 17.77)	15.75 (12.97 – 18.54)	26.42 (23.13 – 29-71)
Municipality				
Bucaramanga	469 (46.30)	12.24 (9.21 – 15.27)	15.75 (12.44 – 19.07)	29.93 (25.87 – 34.00)
Floridablanca	313 (30.90)	16.84 (10.61 – 23.08)	21.21 (14.52 – 27.89)	26.42 (21.68 – 31.16)
Girón	97 (9.58)	31.63 (13.42 – 49.84)	18.54 (3.33 – 33.75)	35.51 (26.37 – 44.65)
Piedecuesta	128 (12.64)	16.60 (0.37 – 32.83)	21.81 (4.19 – 39.44)	28.36 (20.92 – 35.79)
Other	6 (0.59)	-	-	-
Socioeconomic status				
1 (lowest)	69 (6.81)	19.51 (10.29 – 28.73)	18.06 (9.00 – 27.11)	27.51 (16.40 – 38-62)
2	204 (20.14)	22.18 (16.59 – 27.77)	18.42 (13.12 – 23.72)	36.48 (30.26 – 42.70)
3	354 (34.95)	16.24 (12.41 – 20.00)	18.78 (14.78 – 22.79)	28.48 (23.88 – 33.08)
4	262 (25.86)	12.96 (8.84 – 17.09)	17.33 (12.80 – 21.86)	28.96 (23.62 – 34.31)
5	66 (6.52)	8.00 (0.99 – 15.00)	22.42 (12.56 – 32.28)	19.15 (10.00 – 28.24)
6 (higher)	54 (5.33)	12.36 (3.42 – 21.30)	16.84 (6.92 – 26.77)	17.33 (6.59 – 28.07)
Unknown	4 (0.39)	-	-	-
Occupational sector**				
Health	542 (53.50)	14.30 (11.34 - 17.26)	20.60 (17.26 - 23.94)	17.21 (14.06 - 20.36)
Public transportation	31 (3.06)	11.87 (0.41 - 23.33)	23.15 (8.84 - 37.45)	53.45 (36.16 - 70.74)
Military and law enforcement	35 (3.46)	30.06 (13.80 - 46.31)	25.93 (10.37 - 41.50)	25.93 (10.37 - 41.50)
Cleaning	12 (1.18)	27.0 (2.53 - 51.53)	47.15 (19.26 – 75.03)	37.09 (10.42 - 63.75)
Informal commerce	1 (0.10)	-	-	57.33 (0 - 100)
Bike couriers	1 (0.10)	-	-	-
Construction	110 (10.86)	16.48 (9.58 - 23.38)	12.12 (5.89 - 18.34)	46.30 (37.11 - 55.49)
Education	13 (1.28)	37.09 (6.30 - 67.88)	23.63 (0 - 50.78)	64.00 (31.53 - 96.46)
Self-employed worker	12 (1.18)	27.03 (5.81 - 48.24)	27.03 (5.81 - 48.24)	34.54 (11.84 - 57.24)
Food	30 (2.96)	10.90 (0.10 - 21.71)	7.39 (0 - 16.91)	42.90 (26.58 - 59.23)
Security	6 (0.59)	18.66 (0 - 41.41)	29.69 (3.39 – 55.99)	62.78 (33.35 - 92.21)
Bank services	28 (2.76)	9.81 (0 - 19.81)	9.81 (0 - 19.81)	42.54 (26.92 - 58.16)
Administrative municipal services	31 (3.06)	27.87 (13.40 - 42.35)	31.27 (16.31 - 46.22)	55.51 (38.95 - 72.07)
Public utilities	62 (6.12)	9.69 (1.60 - 17.79)	7.51 (0.10 – 14.97)	44.24 (31.45 – 57.02)
Other	99 (9.77)	22.06 (13.20 - 30.91)	11.63 (4.5 - 18.78)	34.06 (24.00 - 44.11)

*Median (IQR). **This information is updated according to the answers for the last round of recruitment. CI corresponds to Confidence Intervals for proportions

**Table 2:** Clinical characteristics and exposure to SARS-CoV-2 by seropositivity across rounds

Variable	All n=1013 n%	Seropositivity		
		Round 1 % (95% CI)	Round 2 % (95% CI)	Round 3 % (95% CI)
Smoking status				
Yes (current smoker)	37 (3.65)	7.03 (0 – 16.01)	4.96 (0 - 13.08)	29.45 (15.14 - 43.75)
Yes (former smoker)	168 (16.58)	12.60 (7.50 - 17.70)	18.18 (12.41 – 23.95)	33.45 (26.50 - 40.40)
Yes (passive smoker)	53 (5.23)	14.66 (5.10 - 24.22)	16.48 (6.54 - 26.42)	31.03 (18.90 - 43.15)
No	755 (74.53)	17.33 (14.65 – 20.00)	19.03 (16.26 - 21.79)	28.12 (24.99 - 31.24)
Medical conditions				
Yes	218 (21.52)	14.18 (11.72 – 16.63)	17.57 (14.93 – 20.21)	23.51 (20.61 – 26.41)
No	795 (78.48)	16.60 (11.78 – 21.42)	23.51 (18.11 – 28.91)	28.48 (22.76 – 34.20)
COVID-19-related symptoms since March 2020				
Yes	223 (22.01)	38.30 (34.86 – 41.74)	44.60 (41.06 – 48.14)	39.03 (35.57 – 42.48)
No	790 (77.99)	9.69 (6.09 – 13.29)	8.36 (4.93 – 11.79)	24.12 (19.24 – 28.99)
Positive RT-PCR results since March 2020				
Yes	291 (28.73)	26.18 (21.25 - 31.10)	35.39 (30.03 - 40.74)	53.69 (47.96 - 59.43)
No	477 (47.09)	10.42 (7.58 - 13.26)	11.15 (8.24 - 14.05)	18.54 (15.09 – 21.99)
Do not know	31 (3.06)	31.87 (15.90 - 47.85)	28.00 (12.59 - 43.40)	31.87 (15.90 - 47.85)
Not applicable*	214 (21.13)	-	-	-

*Participants had never undergone RT-PCR testing for COVID-19. CI = Confidence Intervals for proportions

**Table 3:** Incidence and recurrence of seropositivity in rounds 2 and 3

Variable	Frequencies							
	IgM or IgG		Only IgM		Only IgG		IgM and IgG	
	n	% (95%CI)	n	% (95%CI)	n	% (95%CI)	n	% (95%CI)
SI 1	84	8.60 (2.24 -14.96)	2	0 (0 – 4.67)	55	4.72 (0 – 11.28)	25	0.24 (0 – 6.82)
SPR 1	98	8.36 (2.53 – 14.19)	30	0.2 (0 – 6.24)	85	6.78 (0.92 – 12.65)	18	0 (0 – 4.75)
SI 2	187	26.18 (20.03 - 32.32)	45	5.33 (0 – 12.83)	120	17.33 (10.61 – 24.05)	22	0.12 (0 – 7.01)
SPR 2	69	4.96 (0 – 10.90)	16	0 (0 – 4.50)	43	1.81 (0 – 7.81)	2	0 (0 – 3.16g)

A total of 161 participants were seropositive in Round 1 (52 positive for IgM/IgG, 27 for only IgM, 82 for only IgG). These individuals were excluded from the denominator when calculating seroincidence, as they were not considered part of the population at risk.

**Figure 2a:**
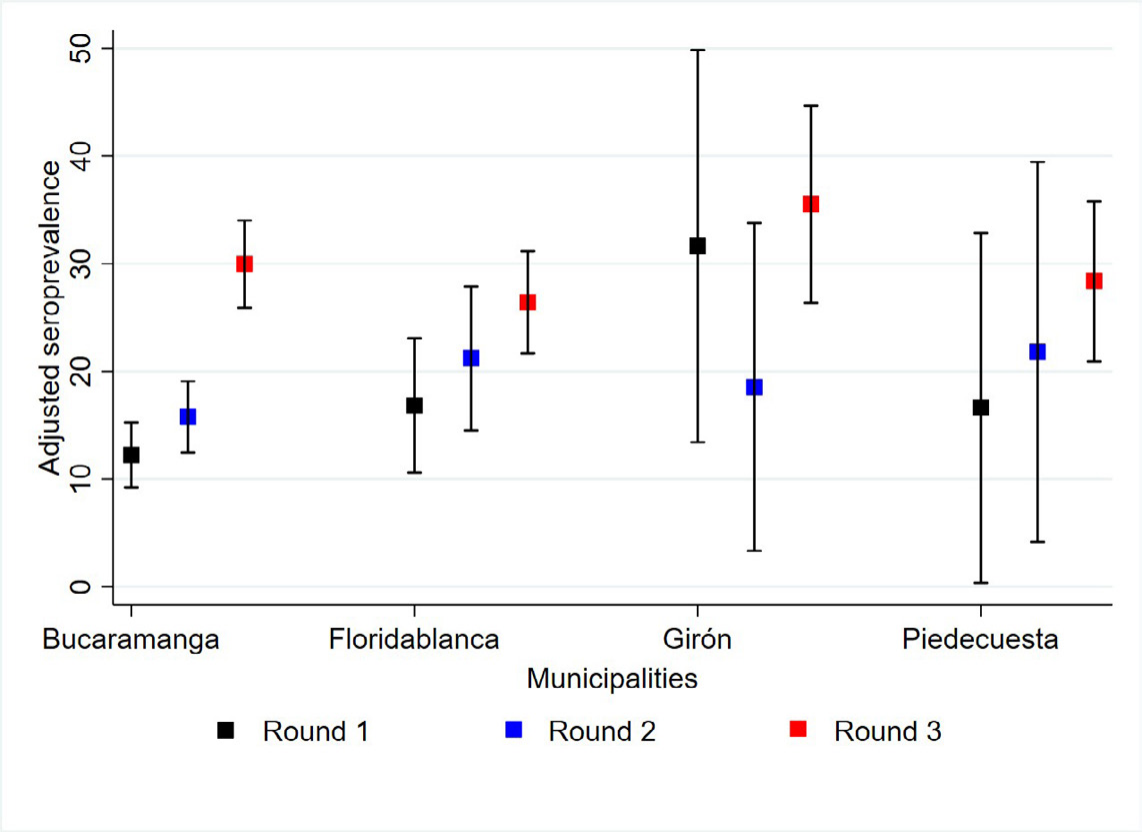
Adjusted seroprevalence by municipality

**Figure 2b:**
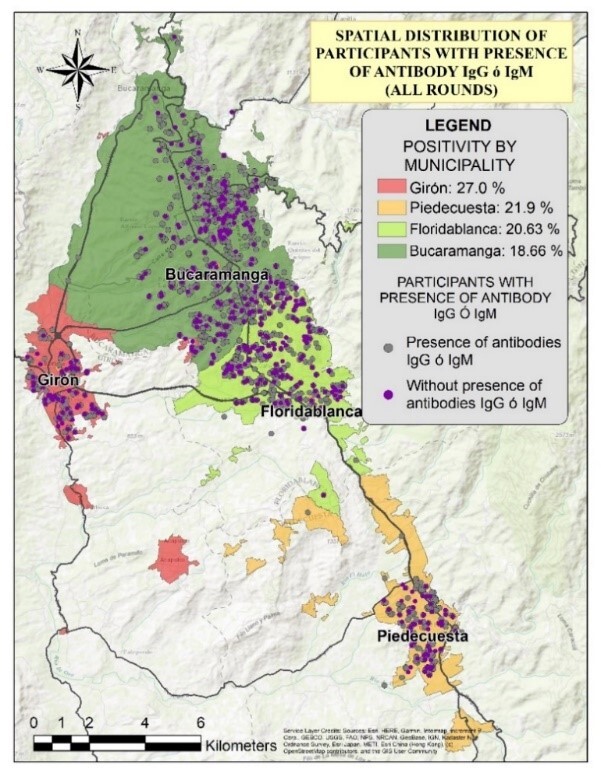
Spatial distribution according to seropositivity

 Figure 2. Adjusted Seroprevalence Increase Across Rounds and Municipalities

*Note: The proportions shown in the figure correspond to crude seroprevalence values*

**Figure 3:**
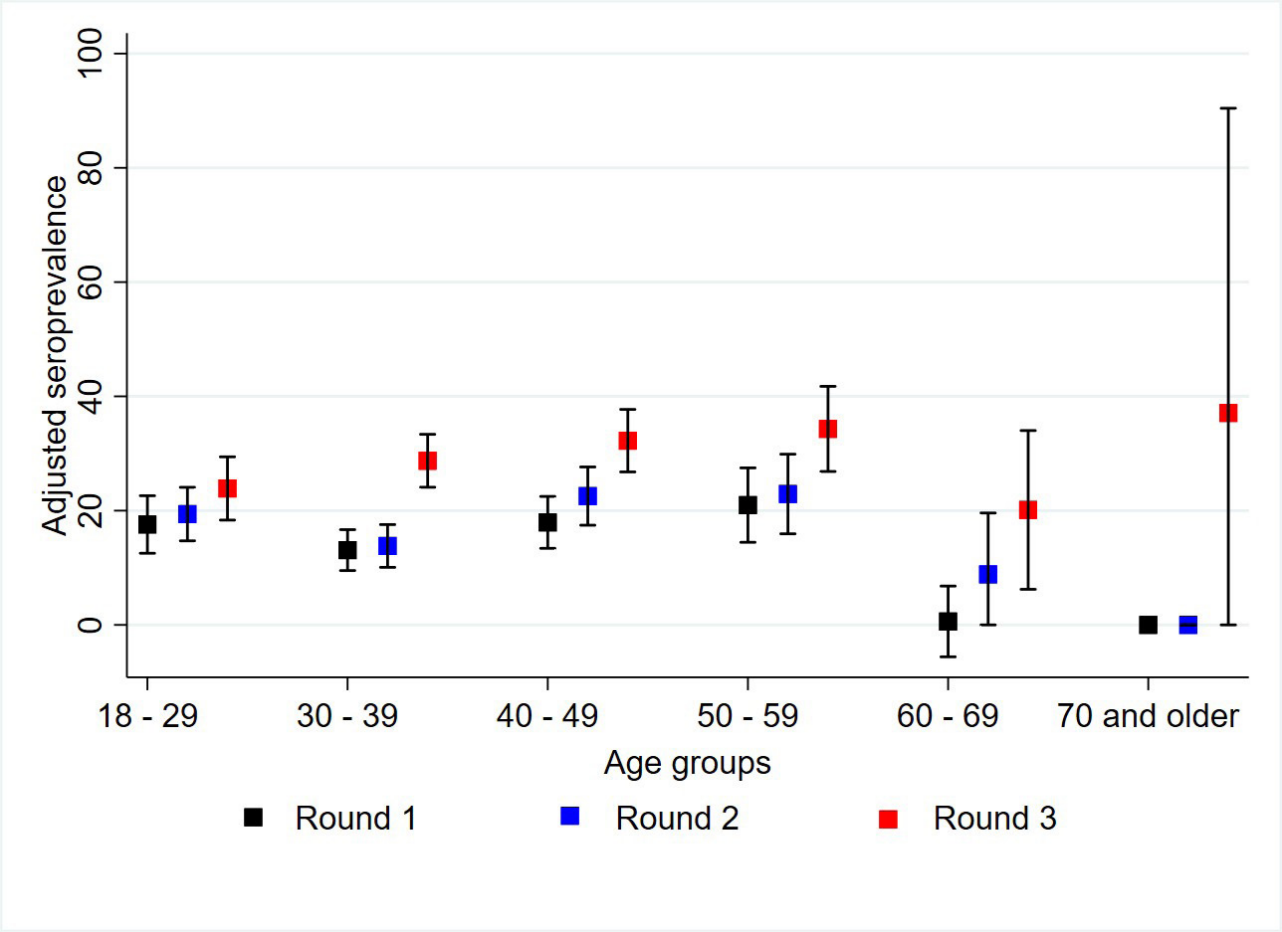
Adjusted seroprevalence by age group

**Figure 4:**
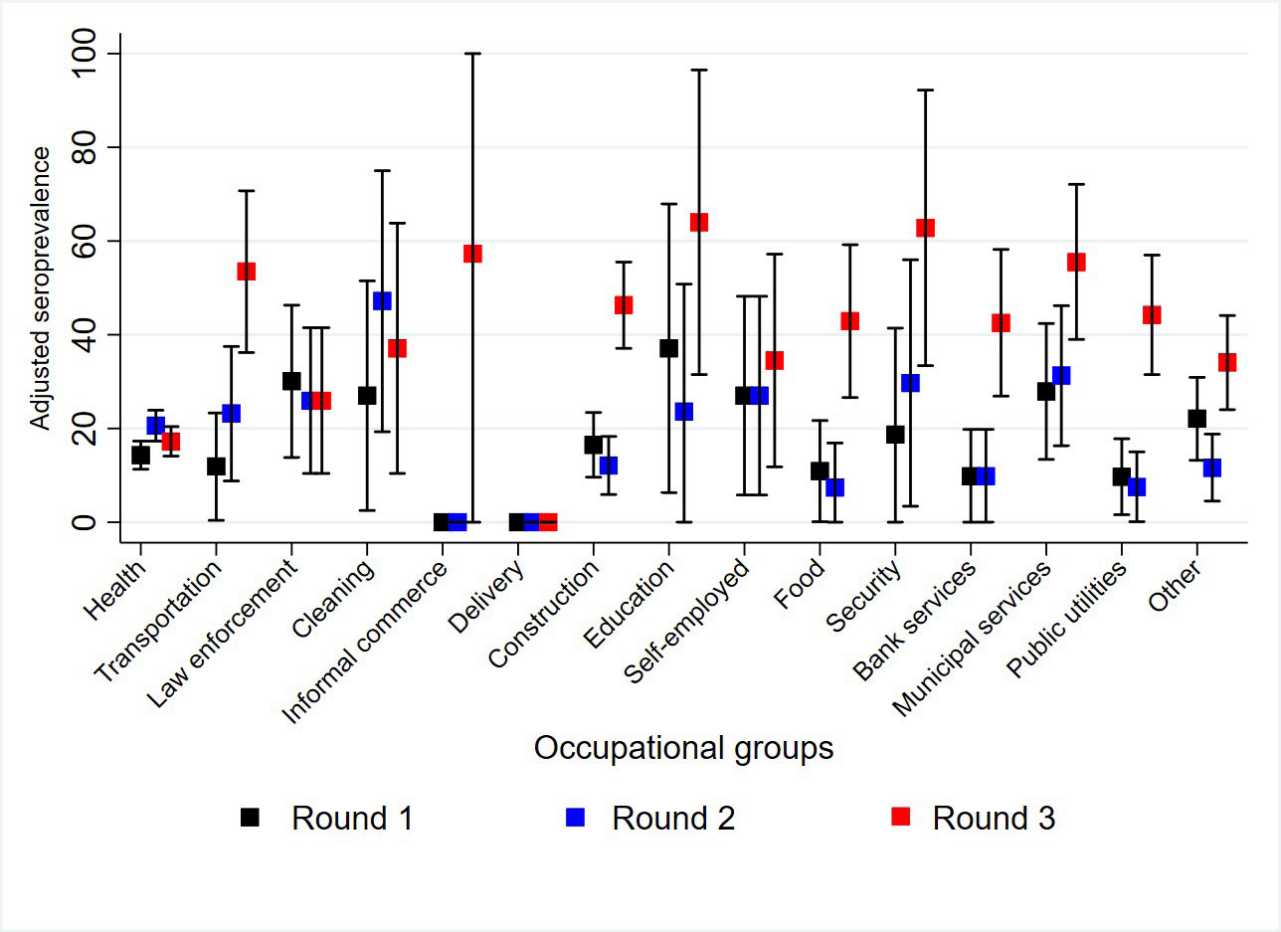
Adjusted seroprevalence by occupational group

## Discussion

The results presented here are the only data available in Colombia derived from the same population with three consecutive measurements of IgM and IgG antibodies, corresponding to the first three waves of the pandemic. Therefore, this study constitutes the most robust available approach to tracking the evolution of the pandemic in Colombia using antibodies. All findings should be interpreted in light of some facts of the pandemic, as described in previous studies^17^,^18^. 

Among the findings, it was evident that men consistently showed higher infection than women. This pattern was expected since existing data from Santander and Colombia indicate that the male sex has been associated with an increased risk of COVID-19 mortality^7^,^18^,^19^. In Bucaramanga, Floridablanca, and Piedecuesta, the occurrence increased progressively across rounds. In contrast, Girón exhibited greater occurrences in the first and third rounds, which can be attributed to weaker public health surveillance capacity^16^, higher social vulnerability, and higher levels of informal employment. The SARS-CoV2 infection tended to be less frequent among workers with higher socioeconomic status, a trend that had already been reported in previous studies^7^,^18^,^19^. 

Occupational groups had different infection profiles during the three rounds. Education sector workers exhibited high infection rates in the three rounds, although the number of participants in this group was very small. People working in the military and law enforcement, cleaning services, and municipal administrative services had the highest infection rates across the three rounds. It was noteworthy that workers in public transportation, construction, private security, food services, public utilities, municipal administration, and banking services had the highest infection rates in the third round. This trend may be related to increased economic activity at the end of the year and the beginning of the new year. 

An interesting finding is the proportion of seropositive participants who reported never having experienced symptoms. This proportion increased progressively across study rounds. Something similar happened among participants who reported never having tested positive for RT-PCR. These values can serve as a proxy for the magnitude of asymptomatic infection, which remains one of the most difficult indicators to quantify and a major limitation to obtaining complete knowledge of the scale of infection during the pandemic. 

This study has some limitations that should be considered when interpreting the findings. The most important is that the results are not representative of any specific population group. Health workers constituted the largest occupational group in the sample; therefore, the estimations for this group are the most robust. For other occupation categories, the estimates should be interpreted as indicative trends of infection occurrence. This limitation is largely due to the voluntary nature of participation, which could overrepresent workers who sought to be better cared for or employed by companies more engaged in monitoring employee health conditions. 

On the other hand, it is important to remember that studies based on IgM and IgG antibody detection tend to underestimate the extend of infection. This is because the duration of detectable antibody responses is shorter in asymptomatic individuals or those with mild symptoms (approximately 30 days). In addition, positive IgM results tend to last less time than positive IgG results^20^,^21^. Our results on the incidence of SARS-CoV-2 seropositivity and seropositivity recurrence were consistent with this immunological dynamic. The marked increase in seroincidence between Rounds 2 and 3 suggests that the infection occurred by a different variant of SARS-CoV-2. 

This study offers valuable insights into the dynamics of SARS-CoV-2 infection among occupational groups in the Bucaramanga Metropolitan Area during three distinct periods of the pandemic. The findings reveal a progressive increase in adjusted seroprevalence, indicating a significant spread of the virus within the population studied. 

Despite widespread vaccination efforts, the study demonstrates the relevance of hybrid immunity (the combined effect of natural infection and vaccination) in shaping immune protection. These results underscore the need for ongoing surveillance and targeted interventions, particularly for vulnerable occupational groups. Informal workers, who often lack access to healthcare services and preventive resources, face heightened risks of infection and complications. Strengthening health policies that provide universal healthcare coverage, social support systems, and workplace safety regulations for informal laborers is crucial for mitigating pandemic-related health disparities. 

Future research should focus on longitudinal monitoring of infection rates and immunity trends across occupational groups, evaluate the efficacy of policy interventions, and investigate socioeconomic determinants of COVID-19-related disparities. Additionally, exploring the influence of political decisions and social events on public perceptions, assessing the long-term impacts of lockdown measures, and examining cross-sectoral collaboration for pandemic preparedness and health system resilience are crucial for informing comprehensive strategies to address future health crises. 

## Conclusions

The study provides unique insights into the dynamics of SARS-CoV-2 infection among occupational groups in the Bucaramanga Metropolitan Area across successive pandemic waves. The results highlight a progressive increase in adjusted seroprevalence of antibodies, particularly among healthcare workers, reflecting their higher exposure and vulnerability. Despite increasing vaccination efforts, hybrid immunity (resulting from previous infections combined with vaccination) continues to play a crucial role in shaping infection rates. This finding underscores the importance of ongoing monitoring and targeted interventions, especially for informal workers who remain at increased risk due to limited access to preventive measures and healthcare resources. In terms of public health and occupational health, this study reveals the need for adaptive and context-specific strategies to protect the most vulnerable populations, ensuring a more equitable and effective response to future epidemiological threats. 

## Figures and Tables

*This density map enables the identification of urban sectors with the highest number of participants, expressed as participants per square kilometer within each municipality of the metropolitan area.*

*The highest participant density was observed in the eastern sector of Bucaramanga across all study rounds, with a density of up to 159 participants per square kilometer. Lower-concentration areas were identified in the southern sector of Floridablanca, followed by Piedecuesta, and finally, Girón, where participant distribution was more dispersed across the urban area. *

*Based on detailed georeferencing, the number of participants was obtained for each municipality within the metropolitan area. Bucaramanga had the highest number of participants across all rounds, followed by Floridablanca, Piedecuesta, and Girón, with the lowest number of participants in all study rounds.*
